# Plant-Derived Compounds in Hemp Seeds (*Cannabis sativa* L.): Extraction, Identification and Bioactivity—A Review

**DOI:** 10.3390/molecules30010124

**Published:** 2024-12-31

**Authors:** Virginia Tanase Apetroaei, Daniela Ionela Istrati, Camelia Vizireanu

**Affiliations:** Faculty of Food Science and Engineering, Dunarea de Jos University of Galati, 111 Domneasca Street, 800201 Galati, Romania; virginia.ape7@gmail.com (V.T.A.); camelia.vizireanu@ugal.ro (C.V.)

**Keywords:** *Cannabis sativa* L., extraction, terpenes, phenolic compounds, cannabinoids, biological activity, health benefits

## Abstract

The growing demand for plant-based protein and natural food ingredients has further fueled interest in exploring hemp seeds (*Cannabis sativa* L.) as a sustainable source of and nutrition. In addition to the content of proteins and healthy fats (linoleic acid and alpha-linolenic acid), hemp seeds are rich in phytochemical compounds, especially terpenoids, polyphenols, and phytosterols, which contribute to their bioactive properties. Scientific studies have shown that these compounds possess significant antioxidant, antimicrobial, and anti-inflammatory effects, making hemp seeds a promising ingredient for promoting health. Since THC (tetrahydrocannabinol) and CBD (cannabidiol) are found only in traces, hemp seeds can be used in food applications because the psychoactive effects associated with cannabis are avoided. Therefore, the present article reviews the scientific literature on traditional and modern extraction methods for obtaining active substances that meet food safety standards, enabling the transformation of conventional foods into functional foods that provide additional health benefits and promote a balanced and sustainable diet. Also, the identification methods of biologically active compounds extracted from hemp seeds and their bioactivity were evaluated. Mechanical pressing extraction, steam distillation, solvent-based methods (Soxhlet, maceration), and advanced techniques such as microwave-assisted and supercritical fluid extraction were evaluated. Identification methods such as high-performance liquid chromatography (HPLC) and mass spectrometry (MS) allowed for detailed chemical profiling of cannabinoids, terpenes, and phenolic substances. Optimizing extraction parameters, including solvent type, temperature, and time, is crucial for maximizing yield and purity, offering the potential for developing value-added foods with health benefits.

## 1. Introduction

**Figure 1:**
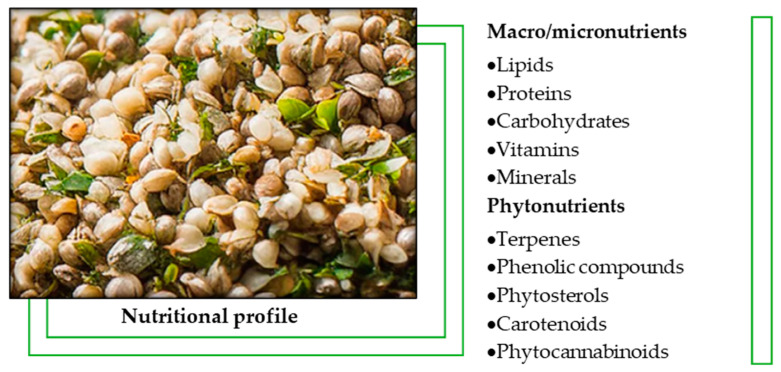
Nutritional composition of hemp seeds.

Hemp seeds (*Cannabis sativa* L.) have significant nutritional and pharmacological value [1,2,3], being an important source of macronutrients, micronutrients, and phytochemicals (Figure 1). The main constituents of the seeds include easily digestible proteins (20–25%), such as edestin and albumin, which contain all essential amino acids and are suitable for both human and animal consumption. Hemp seeds are rich in polyunsaturated fatty acids (PUFA) (25–35%), especially linoleic acid (LA, omega-6) and alpha-linolenic acid (ALA, omega-3), in an optimal 3:1 ratio for human nutrition. Additionally, the seeds contain a significant proportion of carbohydrates (20–30%), most of which are insoluble fibers essential for digestive health [4,5].

The seeds are also a rich source of natural antioxidants and bioactive compounds, including phenolic compounds, bioactive peptides, phytosterols, carotenoids, and tocopherols, contributing to their complex biological activity. The phytochemicals in *Cannabis sativa* L. seeds exert their medicinal properties depending on their concentrations, stability, volatility, and physico-chemical parameters, having varied pharmacological actions. The bioactive compounds in hemp seeds, such as cannabinoids, terpenoids, and phenolic compounds, have demonstrated therapeutic and functional properties, generating increasing interest in food and pharmaceutical research [6,7].

Exploring hemp seed compounds has become an increasingly active research area due to their potential for food and therapeutic applications.

In the past decade, hemp seeds have been included as functional ingredients in a wide range of foods, beverages, nutritional supplements, alternative protein sources, and pharmaceutical products due to the strong biological activity of their metabolites. Including hemp flour in cakes, bread, and pasta can increase the content of phenolic compounds, antioxidant activity, ash, proteins, and fats. Hemp milk may be an alternative to cow’s milk, providing high-quality plant proteins, beneficial fats, and essential minerals. It has been shown to have antithrombotic, anti-vasoconstrictive, anti-inflammatory, and neuroprotective properties, as well as the ability to reduce vomiting. Extracts from *Cannabis sativa* L. seeds exhibit significant antimicrobial activity against foodborne pathogens in meat, making them promising natural preservatives for the food industry [8,9,10].

Cannabinoids, like cannabidiol (CBD), have been extensively studied for their anti-inflammatory and antioxidant properties [11]. Terpenes, volatile aromatic compounds, are known for their antibacterial, antifungal, and anxiolytic effects [12,13], while phenolic compounds are noted for their antioxidant effects that combat oxidative stress [3]. Understanding these compounds and their bioactive potential is essential for evaluating their use in the food industry and contributing to creating functional foods with health benefits [14].

A key aspect of harnessing these compounds is the appropriate selection of extraction methods. The purpose of extracting phenolic compounds, cannabinoids, and terpenes from hemp seeds (*Cannabis sativa* L.) is to obtain valuable ingredients and harness their bioactive and therapeutic potential for the production of dietary supplements, cosmetics, drugs, pharmaceutical products, and functional foods aimed at supporting human health [6,11,12]. The extraction process directly influences the efficiency and purity of the obtained compounds, with the choice of method depending on factors such as the target compounds, extraction conditions, and solvent used [15]. Traditional methods, such as mechanical pressing and solvent extraction, are widely used [16,17]. However, the extraction of cannabinoids and other bioactive compounds from hemp seeds has gained significance, with modern extraction techniques such as supercritical fluid extraction (SFE), microwave-assisted extraction, and sonication demonstrating efficiency, selectivity, and producing high-quality extracts [18,19]. Supercritical CO_2_ extraction offers an eco-friendly approach, enhancing extract quality and reducing contamination risk [20]. This method also provides a safe separation approach, essential in today’s food industry [21,22].

The identification and characterization of phenolic compounds, cannabinoids, and terpenes after extraction from hemp seeds (*Cannabis sativa* L.) allow for the evaluation of their chemical composition, determination of concentration and purity—essential information for optimizing their use in pharmaceutical products, dietary supplements, cosmetics, and functional foods. This process is crucial for ensuring the quality and safety of products intended to support human health [3,4,7,23].

Given the ongoing advances in hemp seed research, a systematic examination of this plant’s nutritional, technological, and functional aspects is essential. Therefore, the present article reviews the scientific literature on traditional and modern extraction methods of bioactive compounds from hemp seeds, their identification methods, and bioactivity. Various extraction methods are reviewed based on their ability to obtain valuable compounds from hemp seeds. Extraction techniques include cold pressing, Soxhlet extraction, and supercritical CO_2_ (SC-CO_2_) extraction, as well as static and dynamic maceration methods, microwave extraction and other processes. Each method is evaluated both for specific operating parameters and for its effectiveness. Extraction conditions vary considerably between methods, including parameters such as temperature (between 40–180 °C), pressure (300–400 bar) and extraction time (2–6 h), as well as pre-extraction treatments.

This review’s novelty lies in evaluating biologically active compounds from hemp seeds regarding bioactivity, efficiency, and quality, focusing on their antioxidant, anti-inflammatory, antimicrobial, and neuroprotective capacities. These capacities can contribute to transforming food products into functional foods that offer beneficial biological effects on human health. This review is also relevant due to the interest in developing healthy, nutritionally superior food products, exploring opportunities to integrate hemp seeds into food processing, and promoting a balanced and sustainable diet.

## 2. Extraction Techniques for Valuable Hemp Seed Compounds

The present section of the review will discuss the techniques used to extract valuable compounds (terpenes, cannabinoids, phenolic compounds) from hemp seeds using various extraction methods and chromatographic techniques [24,25]. The extraction process for bioactive compounds can be performed using conventional or advanced methods (Figure 1). The efficiency of these methods for obtaining ingredients usable in food and health applications depends heavily on parameters such as the plant variety, seasonal and growth conditions, and the chosen solvent [15]. To optimally harness the cannabinoid, terpene, and flavonoid compounds from industrial hemp seeds (*Cannabis sativa* L.), a detailed understanding of their chemical profile is essential. Understanding these aspects is crucial to assessing the potential use of these compounds as food ingredients or therapeutic agents in various applications [3,6].

Thus, high-quality extraction processes with elevated purity become a key stage, significantly impacting the exploitation, identification, and bioactivity of components present in hemp seeds (*Cannabis sativa* L.) intended for the food industry. Through their bioactive properties, these compounds enable the creation of value-added foods that provide a wide range of health benefits [14,23,26].

### 2.1. Conventional and Advanced Extraction Methods

#### 2.1.1. Mechanical Pressing Extraction

Mechanical pressing is a solid–liquid method used to extract oils from seeds and is classified into hot pressing (above 49 °C) and cold pressing (at or below 49 °C). Although simple and environmentally friendly, this method is not widely used for hemp seeds due to their variable maturity, which affects oil yield and quality. Despite its low cost and lack of solvent use, the yield and quality of the product may be influenced by technical parameters and seed pretreatment [16,20].

#### 2.1.2. Steam Distillation and Hydrodistillation

Steam distillation (SD) and hydrodistillation (HD) are used to extract terpenes and cannabinoids by lowering the boiling point of the compounds. Hydrodistillation is more efficient at obtaining higher amounts of bioactive compounds, such as caryophyllene and cannabidiol [12,21].

#### 2.1.3. Solvent-Based Extraction

The Soxhlet method is a standard for solid–liquid extraction and is widely applied to the extraction of bioactive compounds. Using ethanol as a solvent offers flexibility in adjusting extraction parameters, although high temperatures can lead to the degradation of certain compounds like THC [27,28]. Both static and dynamic maceration, whether conducted at warm or room temperature, are highly effective methods for extracting cannabinoids and phenolic compounds. Olive oil can reduce chlorophyll extraction and cannabinoid degradation while using ethanol in dynamic maceration, achieving a higher yield than other methods [14,23]. Microwave-assisted extraction (MAE) uses microwave energy to facilitate thermal transfer and speed up the extraction of cannabinoids, allowing for efficient and controlled extraction while reducing process time [17,18]. Ultrasound-assisted extraction (sonication) enhances the extraction of bioactive compounds while preserving product quality. Sonication is a quick and effective method, often combined with ethanol for extracting compounds from hemp seeds [17,19].

#### 2.1.4. Supercritical Fluid Extraction (SFE)

**Figure 2:**
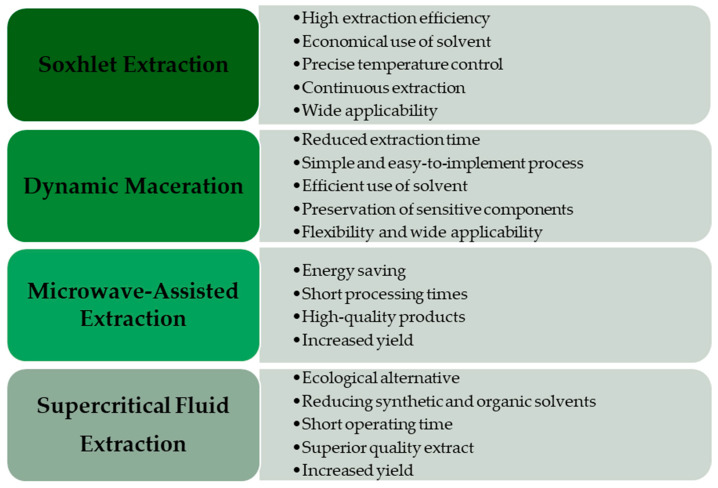
Advantages of advanced extraction methods for valuable hemp seed compounds compared with conventional methods [14,17,21,23].

Supercritical fluid extraction (SFE) is an environmentally friendly method that uses supercritical carbon dioxide to extract compounds from seeds, offering an efficient and selective technique. Figure 2 presents the advantages of using the supercritical fluid extraction method compared with conventional methods. This advanced method minimizes contamination risk and is ideal for obtaining high-quality hemp seed extracts [21].

### 2.2. Purification

The obtained extracts generally contain a wide variety of different compounds. Excess impurities can significantly influence physiological activity, as well as the stability and quality of the final product. Some of these impurities may even be harmful to human health, which makes direct use—mainly in food products—detrimental to quality. Therefore, the purification process becomes essential to ensure the safety of using these extracts as food ingredients. Additionally, higher purity is required for precise addition to foods, enhancing their health benefits and functionality. Sample preparation for determining and identifying fatty acids, terpenes, phenolic compounds, or cannabinoids involves three significant steps: extraction, purification, and identification, as shown in Figure 2 [1,29,30].

### 2.3. Methods for Identifications of Hemp Seed Compounds (Cannabis sativa *L.*)

**Figure 3:**
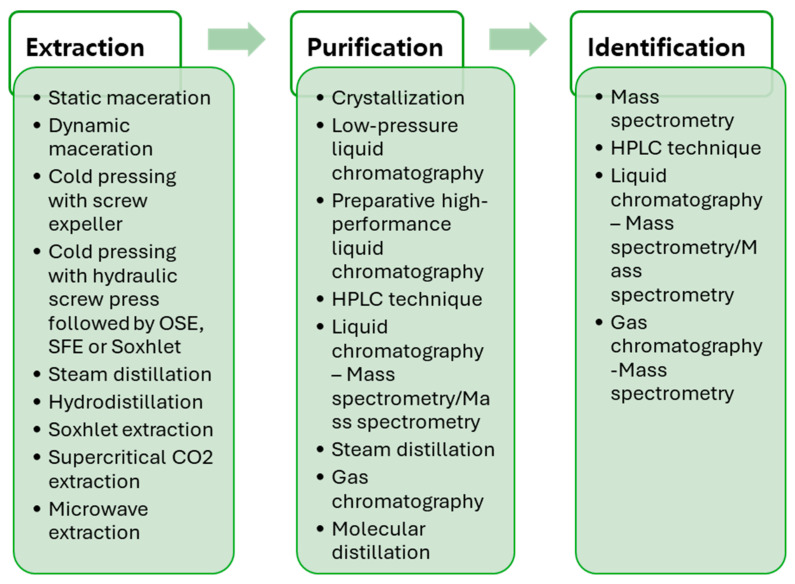
Techniques used in the extraction, purification, and identification of bioactive compounds [32,33,34,35].

High-performance liquid chromatography (HPLC) and mass spectrometry (MS) are used to identify and quantify bioactive compounds, including terpenoids and cannabinoids. These methods provide precise separation and detailed analysis of bioactive components in hemp seeds, aiding in understanding their potential for food and therapeutic applications [31,32]. Figure 3 presents bioactive compounds’ extraction, purification, and identification methods from hemp seeds (*Cannabis sativa* L.).

Table 1 summarizes the extraction methods for bioactive compounds and fatty acids extracted from hemp seeds (*Cannabis sativa* L.), including the solvents used, special conditions applied, and the advantages/disadvantages of each method. Each method has specific applicability depending on the target compounds and the chosen solvent. The selection of the optimal technique depends on the process requirements and the desired chemical composition of the final product.

## 3. Extraction Techniques for Bioactive Compounds in Hemp Seeds (*Cannabis sativa* L.)

### 3.1. Techniques of Extracting Fatty Acids from Hemp Seeds

Before the extraction of fatty acids, hemp seeds (*Cannabis sativa* L.) are often subjected to chemical and physical treatments to improve the extraction yield and obtain a higher-quality oil. These treatments may include cleaning to remove impurities such as dust, dirt or chemicals used in conventional agriculture, decontamination to prevent the growth of bacteria or molds during the extraction process, treatment with solvents such as hexane, due to its ability to dissolve lipids, followed by distillation to obtain a final product that is clean and safe for consumption [20,36], mechanical pretreatment by which hemp seeds can be ground or pressed partly to facilitate the release of fatty acids [37], dehulling, which removes the husk, mechanically or chemically (using mild chemicals) to obtain a clean product, or enzyme treatment that helps break down the cellular structures of the seed, which can enhance the efficiency of the extraction process. Less-invasive methods, such as cold pressing, which do not involve chemical solvents, are preferred in the industry to maintain the quality of the final product.

The extraction of fatty acids from hemp seeds is crucial due to the high nutritional value of omega-6 and omega-3 fatty acids. The ideal ratio of *ω*6:*ω*3, of 3:1, indicates the quality of the obtained oil [16]. In the extraction procedure established by Montero et al. (2023) [36] and Sainz Martinez et al. (2023) [20], cold pressing with a screw press is used to extract oil without solvents. The samples were pretreated by cleaning and grinding. The operating temperature is set at 60 °C, with a frequency of 20 Hz and a nozzle size of 6 mm (Table 1). The main disadvantage of this method is the high amount of pigments extracted, which affects the aesthetic quality of the oil.

Valizadehderakhshan et al. (2021) [37] described the procedure of cold pressing with a hydraulic screw press followed by organic solvent extraction (OSE), supercritical fluid extraction (SFE), or Soxhlet (only on a small scale), using either polar or nonpolar solvents. The sample pretreatment (crushing and grinding the seeds into smaller pieces) contributes to improved mass transfer under the concentration gradient conditions between the saturated solution in contact with the seed particles and that of the environment.

In another protocol, Lopez (2020) [27] used the Soxhlet method with n-hexane as a solvent, and the extraction took place at 180 °C for 2 h and 45 min. Although the yield is good, the long extraction time and the large amount of solvent used are disadvantages related to toxicity and the need to remove residual solvent from the final product. The use of n-hexane also raises an additional issue related to the decarboxylation of acidic cannabinoids at high temperatures, which is to convert them into neutral forms such as THC and CBD, which can affect the biochemical profile and therapeutic properties of the extract. Thus, the high temperatures involved in Soxhlet extraction accelerate this decarboxylation, reducing the ability to isolate cannabinoids in their acid form (THCA and CBDA).

Sainz Martinez (2023) [20] and Valizadehderakhshan et al. (2021) [37] describe the SC-CO_2_ extraction method (supercritical CO_2_) as an eco-friendly method that does not involve toxic solvents. Supercritical CO_2_ at 40–80 °C and 300–400 bar pressures over 4–6 h ensures a high-quality oil with optimal fatty acid and tocopherol (vitamin E) content. Compared to cold pressing and the Soxhlet method, this technique produces oil with fewer impurities but involves higher equipment costs.

The introduction of ultrasound into the supercritical CO_2_ extraction process significantly reduces the extraction time while maintaining the superior quality of the oil. The operating conditions are similar to those of the conventional SC-CO_2_ method. However, pretreatment through ultrasound exposure for 10 min increases the efficiency of the process, making it a viable eco-friendly alternative, according to studies presented by Sainz Martinez (2023) [20] and Valizadehderakhshan et al. (2021) [37].

Following the extraction processes, the extracted compounds are identified and characterized. These include omega-6 and omega-3 fatty acids, flavonoids, terpenes, and cannabinoids such as THC and CBD. Mass spectrometry (MS) is an extremely useful analytical technique for characterizing chemical compounds in various matrices, including hemp seeds (*Cannabis sativa* L.). This method can be used to identify and quantify a wide range of bioactive substances. Also, high-performance liquid chromatography (HPLC) analyzes phytocannabinoids, employing a mobile phase of a formic acid and acetonitrile mixture. According to studies by Benkirane et al. (2022) [31], the technique is standardized and offers a clear separation of the main phytocannabinoids (THC and CBD). The retention time is 13–15 min at a temperature of 40 °C. Liquid chromatography–mass spectrometry/mass spectrometry (LC-MS/MS) is utilized for precise quantification of phytocannabinoids, allowing for multi-component analysis by identifying and quantifying phytocannabinoids at low levels, making it an extremely sensitive and accurate method for detecting THC and CBD [35]. Gas chromatography–mass spectrometry (GC-MS) is a very effective laboratory technique used for the identification and quantification of bioactive substances such as terpenes, fatty acids, cannabinoids, and other organic compounds [12,33].

### 3.2. Techniques of Extracting Phenolic Compounds from Hemp Seeds

The extraction procedure for phenolic compounds from hemp seeds, known for their antioxidant and anti-inflammatory properties, as applied by Montero et al. (2023) [36], uses static maceration with ethanol, which is considered eco-friendly and suitable for the food industry. The process takes place at room temperature to preserve the integrity of light-sensitive compounds in olive oil and reduce the extraction of chlorophyll. This intensely colored green compound is undesirable in some food matrices. According to the same study, combining this method with steam distillation increases efficiency [36]. One advantage of this method is the use of olive oil, which ensures the stability and quality of the extract; however, it could also limit the time required for complete extraction. Table 2 presents the flavonoid compounds that can be extracted from hemp seeds (*Cannabis sativa* L.) and used in food products, transforming them into healthy, value-added products with health benefits.

### 3.3. Techniques of Extracting Terpenes from Hemp Seeds

Terpenes are essential bioactive compounds due to their antioxidant, anti-inflammatory, and aromatic properties. The extraction of these compounds requires appropriate methods and solvents to achieve optimal results. Extraction methods such as steam distillation (SD) and hydrodistillation (HD) use water vapor to extract terpenes. In the case of SD, the process occurs at 130 °C, while for HD, it is conducted at 110 °C. Caryophyllene, a significant compound known for its aroma and therapeutic antioxidant and anti-inflammatory properties, is obtained in the initial minutes of extraction. According to studies by Al Ubeed et al. (2022) [23] and Valizadehderakhshan et al. (2021) [38], SD yields better results for extracting monoterpenes (54%) compared to sesquiterpenes (44.2%), while HD extracts a higher amount of sesquiterpenes (48.5%) in relation to monoterpenes (43.9%).

The water vapor used in both SD and HD facilitates the extraction of monoterpenes and sesquiterpenes without introducing additional chemical compounds. This is an eco-friendly method; however, it depends on the temperature and duration of the extraction.

Static maceration involves using a mixture of organic solvents such as ethyl acetate, methanol (MeOH), and chloroform, each solvent having an affinity for certain compounds based on polarity. The process is conducted at room temperature, preserving the chemical structure of the compounds, and combining it with SD can enhance the efficiency of terpene extraction [23,37]. Static maceration is frequently used to extract heat-sensitive compounds, ensuring the terpenes’ integrity is maintained.

Dynamic maceration involves using vegetable oils, grinding the seeds, and immersing the sample in the chosen organic solvent. The extraction of terpenoids is achieved through the appropriate polarity of the solvent used, such as ethanol (EtOH), methanol (MeOH), or chloroform, at a specific temperature and for a defined time [14,23]. Dynamic maceration is a versatile method that allows for the adjustment of extraction parameters to optimize results; however, it requires a longer duration and continuous agitation for maximum efficiency. Table 3 contains some terpenes identified in hemp seeds and their bioactivity (*Cannabis sativa* L.).

### 3.4. Techniques of Extracting Phytocannabinoids from Hemp Seeds

Hemp seeds (*Cannabis sativa* L.) are recognized for their high nutrient content and are widely marketed as health foods. However, in the food industry, monitoring the presence of two major phytocannabinoids associated with this plant, tetrahydrocannabinol (THC) and cannabidiol (CBD), is essential. These compounds belong to the cannabinoid class and are produced and stored in glandular trichomes primarily present on the female plant’s flowers, with fewer numbers on leaves and stems, and are absent on roots and seeds [6,75]. Phytocannabinoids in hemp seeds result from accidental contamination during harvesting through physical contact with the resin secreted by the glandular trichomes on the bracts surrounding the seeds. The level of contamination varies depending on the strain and the seed-cleaning process, and for industrial hemp strains, THC levels should be extremely low [6].

International authorities, including those in Europe and America, use phytocannabinoids concentrations to identify different hemp strains. According to Grassi and McPartland (2017) [76], the THC/CBD ratio is genetically determined, and phytocannabinoids quality can provide a more stable method for classifying cannabis than the absolute phytocannabinoids levels, which can vary due to factors such as the environment and the age of the plant [77,78]. Due to the potential psychoactive effects of THC, many countries have implemented strict regulations regarding the upper limits of THC allowed in hemp-derived food products, highlighting the need for a quantification method for phytocannabinoids contamination in these products. This situation underscores the need for precise analytical methods to monitor phytocannabinoids contamination in hemp seeds and their food derivatives [79,80].

Hemp seeds are analyzed using a series of extraction and analytical methods to quantify phytocannabinoids content (especially CBD and THC), substances known for their therapeutic and psychoactive effects. These methods focus on maximizing extraction efficiency and separating bioactive compounds.

The microwave extraction technique using ethanol (EtOH) involves soaking the seeds in an organic solvent (EtOH), followed by their microwave irradiation. According to the study by Yang et al. (2017) [17], this type of irradiation facilitates rapid and uniform heating, leading to an accelerated extraction of compounds. The efficiency of this method is estimated at η = 27–40% after filtration and evaporation processes, resulting in a significant concentration of phytocannabinoids. However, a major drawback of the method is the conversion of carboxylic acids into neutral forms of THC and CBD at high temperatures, which can affect the composition of the final extract. Sonication, as a method to activate the extraction, involves the application of ultrasonic waves and is frequently used to intensify and accelerate the extraction processes by dissociating the cellular structures, which facilitates better preservation of the integrity of the bioactive compounds [17,39].

Soxhlet (EtOH) is a classic technique in which ethanol continuously circulates through a Soxhlet system at a constant temperature, extracting phytocannabinoids over several reflux cycles. The yield of this process, η = 24–38%, is similar to that of sonication but takes longer. According to the procedures presented by Sander (2017), Yang et al. (2017), and Lazarjani et al. (2021) [14,17,39], Soxhlet is an efficient method for complete extraction; however, at high temperatures, carboxylic acids may be converted into neutral forms.

Supercritical fluid extraction (SFE) with supercritical CO_2_ and EtOH utilizes supercritical CO_2_ combined with ethanol to extract phytocannabinoids from seeds selectively. The yield of 31–37% provides efficient extraction of active compounds. It is an environmentally friendly and energy-efficient method, yielding well compared to Soxhlet and sonication [17].

Static maceration (EtOH) with ethanol and using olive oil for phytocannabinoid protection is an effective method for extracting sensitive compounds. Olive oil protects bioactive phytocannabinoids during extraction [23,37]. Although hemp seeds may contain traces of cannabinoids, their amounts are minimal. In general, hemp seeds are more likely to contain cannabigerol (CBG), but at minimal concentrations. However, extracting active cannabinoids from hemp seeds is not a common practice, given that their levels are insignificant compared to other parts of the plant, such as flowers.

Dynamic maceration uses organic solvents such as n-hexane, acetone, and methanol to extract phytocannabinoids from hemp seeds. According to Lazarjani et al. (2021) [14], alcoholic and hydroalcoholic solvents are effective in extracting a wide range of bioactive compounds, including neutral phytocannabinoids. It is important to note that hemp seeds are not a primary source of cannabinoids compared to other parts of the plant, such as flowers or leaves. Therefore, even with dynamic maceration, the concentrations of cannabinoids extracted from hemp seeds are much lower than those obtained from other parts of the plant. Table 4 presents the phytocannabinoids whose quantification is necessary when using hemp seeds (*Cannabis sativa* L.) in the food industry.

## 4. Optimization

The abundance of bioactive compounds has led to increased interest in the phenolic compounds present in hemp seeds (*Cannabis sativa* L.) due to their antibacterial, anticancer, anti-inflammatory, and antioxidant properties, which derive from their redox characteristics and affinity for proteins and metal ions [7,83]. Additionally, terpenes, which have antibacterial, antifungal, anxiolytic, anti-aging, and antitumor properties, have also gained attention [12,13]. Furthermore, certain terpenes and flavonoids, along with other metabolites, are believed to interact with phytocannabinoids and modify their properties, leading to a variety of pharmacological effects observed in different chemotypes of cannabis. Given all these attributes, optimizing the extraction process and preserving the characteristics of these bioactive compounds becomes particularly important [7,46,65,84].

Choosing an appropriate solvent can influence the extraction efficiency to achieve optimal yields and chemical profiles. Generally, solvents with low density and viscosity are effective in extraction because they have high diffusivity, favoring the movement of solvent and solute molecules, thus improving the efficiency of the extraction process. Phenolic compounds are more soluble in polar solvents, and their solubility levels are influenced by structural characteristics such as molecular size, the presence of hydroxyl groups, the length of hydrocarbon chains, and the degree of methoxylation [3,23]. Carbon dioxide is an economical, safe, non-toxic solvent (leaving no residues in the extract) and quickly reaches supercritical conditions (32 °C and 7.38 MPa). Moreover, CO_2_ is acceptable in the food and pharmaceutical industries [14,85]. Optimizing extraction conditions related to temperature, pressure, and extraction time can be adjusted to maximize the yield and quality of the resulting compounds [86].

Using co-solvents or combinations of solvents can enhance the solubility of compounds and shorten the extraction time required. This conclusion aligns with other research that has indicated the effectiveness of moderately polar mixtures, such as acetone–water, in extracting phenolic compounds from protein-rich plants like hemp seeds (*Cannabis sativa* L.). This efficiency is attributed to the ability of these mixtures to degrade polyphenol–protein complexes [20,23].

Pretreatment techniques such as fine-grinding hemp seeds and applying thermal or enzymatic treatments could enhance extraction efficiency by increasing the contact surface area or breaking down the cell wall [16,87].

Multi-step extraction using different solvents or combining extraction techniques such as Soxhlet or maceration can improve the yield and selectivity of the extraction process. As a result, in the last decade, special attention has been given to developing rapid and precise analytical methods for phytocannabinoid analysis, with minimal sample processing and reduced use of chemicals [25,31].

Optimizing the solvent–plant ratio by adjusting the concentration and volume of solvent relative to the amount of plant material can optimize extraction efficiency and minimize solvent consumption. Combining organic solvents with water can positively impact the extraction of phenolic compounds, as it reduces the density and viscosity of the solvent system, increasing its diffusion and facilitating the extraction process. Adjusting the proportions of solvents can further improve this process. Additionally, the overall phytocannabinoid profile can be influenced by both genetic characteristics and storage conditions [12,35].

Encapsulation of hemp oil, known for its rich content of essential fatty acids and antioxidants, offers significant health benefits but faces the risk of degradation through oxidation during processing, storage, or transportation of food. This issue can compromise the nutritional value and the product’s final quality [7,88]. This deterioration can be prevented by packaging in a modified atmosphere or adding synthetic antioxidants. However, these approaches are associated with certain limitations, including reduced long-term efficacy and concerns regarding the safety and sustainability of the resulting product. Encapsulation, whether in the form of microcapsules or other similar structures with a protective matrix, represents a promising strategy to counteract the harmful effects of environmental factors such as oxygen, light, and humidity, protecting hemp oil from the oxidation process or masking flavor compounds. This technique offers multiple benefits, including extending the oil’s shelf life, improving stability, controlling rapid release and its bioavailability, and providing consumers with a convenient and precise dosing method [89,90]. Consequently, the promising perspective of encapsulation for protecting hemp oil is an impetus for continued research, potentially bringing significant innovations in food systems. These innovations could provide more stable, efficient, and sustainable products for consumer satisfaction. It is emphasized that extraction efficiency is not the only criterion for selecting the appropriate extraction technique, where the cost of extraction is equally important [20,87].

Validation and optimization of precise and sensitive analytical methods for quantifying the obtained results are essential for evaluating extraction efficiency and ensuring the quality of final products [30]. These optimization methods can be applied individually or in combination, depending on the specifics of the extraction process and the analysis objectives [7,14].

## 5. Health Benefits of the Compounds Extracted from Hemp Seeds (*Cannabis sativa* L.)

**Figure 4:**
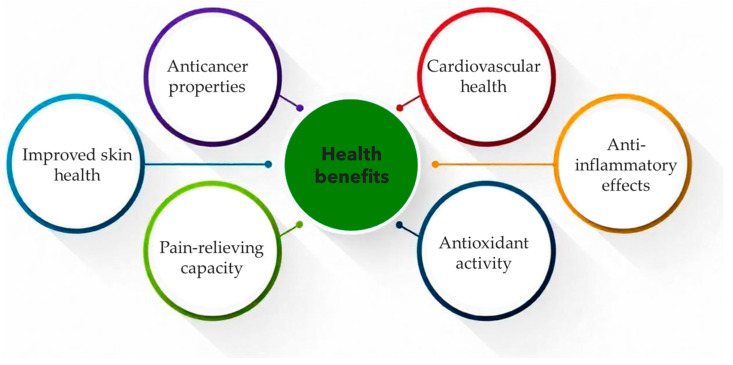
Health benefits of hemp seeds’ bioactive compounds.

In recent years, interest in cultivating hemp (*Cannabis sativa* L.) has significantly increased due to the evidence of the nutritional health benefits of hemp seeds and the oil derived from them (Figure 4). Hemp seed oil contains essential fatty acids and fat-soluble vitamins, which are crucial in maintaining health by supporting physiological functions and nutritional balance [91].

The unsaturated fatty acids in hemp seeds have significant potential for cardiovascular health due to the favorable ratio of unsaturated to saturated fatty acids. Hemp oil, primarily obtained through cold pressing, is characterized by a low content of saturated fatty acids (9.4–11.7%) and a high content of polyunsaturated fatty acids (PUFAs), contributing to an optimal omega-6:omega-3 ratio of 2:1 to 3:1, unlike Western diets, which have a much higher ratio (>15:1) due to the excessive consumption of vegetable oils rich in omega-6, such as sunflower and corn oil [86]. This omega-6/omega-3 balance in hemp seeds may help counteract imbalances in modern diets and reduce the risk of chronic inflammatory diseases associated with a heart-healthy diet [13]. Another important polyunsaturated fatty acid in hemp oil is gamma-linolenic acid (GLA) (1–3%), known for its anti-inflammatory effects, as it is rapidly converted to dihomo-gamma-linolenic acid (DGLA), which has significant biological properties [83].

Additionally, hemp seeds are a valuable source of stearidonic acid (<1%), a precursor for the synthesis of long-chain omega-3 fatty acids, known for their anti-inflammatory effects and cardiovascular benefits [91]. These components contribute to health benefits, including reducing inflammation, alleviating pain, and protecting against cardiovascular diseases [92].

The potential use of hemp seed products (*Cannabis sativa* L.) in human nutrition is determined by their fatty acid composition and their rich content of bioactive compounds, such as polyphenols. Plants produce these as a defense mechanism against pathogens and ultraviolet radiation and have strong antioxidant activity, contributing to the neutralization of free radicals. Thus, these antioxidants help maintain the chemical stability of oil obtained through cold pressing, preventing oxidation and extending its shelf life. In addition to these, other minor components, such as terpenes and cannabinoids, may contribute to the overall benefits of hemp seed products [25].

Hemp seeds (*Cannabis sativa* L.) are a rich source of phytochemical compounds, including polyphenols such as caffeoyltyramine, lignanamides (*cannabisins* A, B, and C), alongside essential amino acids and sugars. Recent studies have shown that hemp seeds contain various bioactive compounds with health benefits, exhibiting antioxidant activity both in vitro and in vivo, as well as antimutagenic activity against the yeast Saccharomyces cerevisiae, suggesting their potential in food applications [12].

The phenolic compounds in seeds are predominantly distributed among three major classes: flavonoids, stilbenes, and lignans. The literature emphasizes that these compounds play a vital role in preventing diseases associated with oxidative stress due to their remarkable antioxidant capacity. The extraction of these molecules without compromising their biological activities has garnered significant interest from researchers [31]. Moreover, canflavins, specific flavonoids found in hemp, have demonstrated notable biological activities. In vivo tests have confirmed their anti-inflammatory and anticancer properties, with these substances having a pain-relieving capacity up to thirty times greater than aspirin [12,31].

Terpenes, structurally, are lipophilic hydrocarbons responsible for the distinctive smell of cannabis, mainly represented by monoterpenes, sesquiterpenes, and triterpenes. These are the main oil compounds that play a crucial role in determining the aromatic and sensory characteristics of the plant. These molecules are recognized for their multiple therapeutic benefits and are primarily used in aromatherapy. Alongside cannabinoids, terpenes contribute to what is known as the synergistic or “entourage” effect, an interaction studied in recent decades that amplifies anti-inflammatory, analgesic, anxiolytic, antibacterial, anticancer, antioxidant, and antifungal potential. Due to these characteristics, terpenes from hemp seeds (*Cannabis sativa* L.) are used in the food and pharmaceutical industry. This aspect makes terpenes promising components for developing functional foods, a rapidly expanding sector [93].

Cannabidiol (CBD) has significant potential for improving skin health and treating dermatological conditions. The endocannabinoid system (ECS) plays an important role in regulating physiological processes at the skin level, suggesting that topical cannabinoid-based treatments could be effective for certain skin disorders or improve skin health. However, most available clinical studies focus on oral, inhaled, or injectable administration of cannabinoids, while research on topical applications is limited. Nevertheless, preliminary evidence suggests that the local use of CBD may represent a viable delivery method for specific skin conditions. For example, the study conducted by Hammell et al. investigated the efficacy of CBD gels (at concentrations of 1–10%) in alleviating inflammatory symptoms associated with conditions such as eczema and atopic dermatitis [94].

In addition to topical effects, CBD has also been shown to have other beneficial functionalities, including pain relief, anxiety and stress reduction, nausea management, and inducing relaxation. Another advantage of using CBD is the absence of psychoactive effects and the potential for addiction, unlike tetrahydrocannabinol (Δ9-THC). With the increasing prevalence of chronic lifestyle-related diseases, there is a growing demand for healthy diets and functional foods that include ingredients with proven benefits. Thus, CBD in functional foods and beverages represents a promising field. While cannabis contains more than 100 cannabinoids, Δ9-THC and CBD dominate discussions in the field due to their psychoactive and medicinal properties, legal status, scientific research, and public recognition. However, as research continues, other cannabinoids will likely receive more attention as their potential benefits are explored. However, significant challenges remain, including legal regulations and technological limitations [11,95,96,97].

## 6. Conclusions

Despite extensive investigations into identifying phytochemical compounds in hemp seeds, there are still unknown or insufficiently characterized substances. This situation complicates a comprehensive assessment of their biological potential and functional applications for developing innovative food products and their acceptability among consumers. There are notable divergences and obstacles in the current literature regarding the phytochemicals derived from hemp seeds (*Cannabis sativa* L.) and their applicative perspectives as functional ingredients in nutrition. These investigations should involve in vitro and in vivo testing to assess their impact on human health. Comparative research is necessary to evaluate the efficacy of phytochemicals from hemp seeds compared to other natural sources. Conducting safety studies to assess potential adverse effects and determine safe and effective doses is essential.

Moreover, the industrial hemp industry (*Cannabis sativa* L.) faces numerous challenges, including restrictive regulations, the risk of exceeding legal THC limits in crops, and technological issues, such as the fragility of planting and harvesting equipment. Additionally, the influence of geographical locations, harvest season, timing of harvest, and cultivation methods on phytochemicals must be considered. Understanding the processes of obtaining these ingredients, influenced by the plant’s variety, seasonality, and growing conditions, becomes essential for fully exploiting the potential of hemp seeds in the food industry.

The need for an interdisciplinary approach and collaboration among experts in chemistry, biology, nutrition, and food technology is evident in addressing these deficiencies and developing a deeper understanding of the bioactive potential of hemp seeds (*Cannabis sativa* L.).

## Figures and Tables

**Table 1:** Methods used to extract biological active compounds from hemp seeds (*Cannabis sativa* L.).

Compounds	Extraction Technique	Solvent	Extraction Conditions/Procedures	Products	Advantages and Limitations	Reference
Fatty acids	Cold pressing with screw expeller	-	The samples were cleaned of impurities and ground using a laboratory mill. Extraction conditions fixed the temperature of the press head at 60 °C, the frequency of 20 Hz, and the nozzle of 6 mm.	The content of fatty acids in hemp oil is similar to that obtained by the Sohlet and SC-CO_2_ method; desirable ratio ω-6:ω-3 (3:1).	The highest total pigment content	[20,36]
	Soxhlet extraction	n—hexane	The samples were cleaned of impurities and ground using a laboratory mill. The extraction conditions were 2 h and 45 min at 180 °C, until the total exhaustion of the fat content.	The fatty acid content of hemp oil is similar to that obtained by the SC-CO_2_ method and cold pressing; it had no tocopherols; desirable ratio ω-6:ω-3 (3:1).	Long extraction time; large amounts of solvent required	[20,36]
	SC-CO_2_	Super critical CO_2_	The samples were cleaned of impurities and ground using a laboratory mill. The extraction conditions were with variations in temperature (40–60 °C), pressure (300–400 bar), and time (4–6 h).	The content of fatty acids in hemp oil is similar to that obtained by the Soxhlet method and cold pressing, but the amount of tocopherols is higher compared to cold pressing; the desirable ratio ω-6:ω-3 (3:1).	The high quality of the product, the absence of solvent in the extracts, the lowest total pigment content	[20,37]
	SC-CO_2_	Super critical CO_2_	Ultrasound of hemp seeds, in the absence of solvent, for 10 min, T = 40–80 °C, P = 200–400 bar	Desirable ratio ω-6:ω-3 (3:1)	Ecological alternative; short operating time; high-quality extract	[20,21]
	Cold pressing with hydraulic screw press followed by OSE, SFE, or Soxhlet (small scale only)	Nonpolar and polar solvents	Crushing and grinding the seeds into smaller pieces contributes to improved mass transfer, ~ (C* − C) = concentration gradient between the saturated solution in contact with the seed particles and that of the environment.	Linoleic fatty acids ω-6 (C_18_H_32_O_2_), oleic ω-9 (C_18_H_34_O_2_)	Linoleic and linolenic acid. SC-CO_2_ selectively extracts acids.	[37]
Flavonoids	Static maceration	EtOH	Olive oil to reduce chlorophyll extraction, at room temperature. Association with SD increases efficiency.	Apigenin, quercetin and luteolin	Effective method: olive oil protects the bioactive compounds.	[34,36]
Terpenes	SD HD	Water vapor	SD (130 °C), HD (110 °C)	*Caryophyllene* is extracted in the first minutes.	SD extracts a higher content of monoterpenes (54%) compared to sesquiterpenes (44.2%); HD extracts sesquiterpenes (48.5%) versus monoterpenes (43.9%).	[23,37]
	Static maceration	Mixture of organic solvents (ethyl acetate, methanol, chloroform/methanol)	Room temperature. Association with SD increases efficiency.	Beta-caryophyllene, myrcene, limonene	The efficiency of the process and the quality of the extracts depend on the solvent chosen	[23,37]
	Dynamic maceration	Vegetable oils	Grinding. Soaking the sample in organic solvents chosen based on the polarity of the target compound at a specific temperature for a specific time, followed by stirring	Beta-caryophyllene, myrcene, limonene, and humulene	Solvents EtOH, MeOH or chloroform extract flavonoids and terpenoids; solvents dichloromethanol, ether, or water extract terpenoids.	[14,23]
Cannabinoids	Microwave extraction	EtOH	Maceration of seeds in mortar with pestle. After maceration, suspension in solvent (ethanol), then heating (150 °C) and stirring (900 rpm/20 min). After cooling (25 °C) and filtering the suspension, it results in a sticky resin used for the analysis and quantification of cannabinoids; η = 27–38%.	Initially, carboxylic acids, Δ9-THCA and CBDA, which are converted to Δ9-THC and CBD by exposing the resin to high temperatures	The estimated amount of Δ9-THC and CBD could be 7 to 12 times higher, respectively, than the legal limit of 10 μg/g of hemp seeds.	[17,38]
	Soxhlet Extraction	EtOH	Maceration of seeds in mortar with pestle. Refluxing with EtOH = 4 h, cooling = 25 °C, results in an oily resin used to analyze and quantify cannabinoids. η = 24–38%.	Initially, carboxylic acids, Δ9-THCA and CBDA, which are converted to Δ9-THC and CBD by exposing the resin to high temperatures	The estimated amount of Δ9-THC and CBD could be 7 to 12 times higher, respectively, than the legal limit of 10 μg/g of hemp seeds. Soxhlet extraction provided higher yields of Δ9-THC.	[14,17,39]
	SFE	Super critical CO_2_ and EtOH	Maceration of seeds in mortar with pestle. Analyze and quantify cannabinoids. η = 31–37%	Initially, carboxylic acids, Δ9-THCA and CBDA, which are converted to Δ9-THC and CBD by exposing the resin to high temperatures	The estimate shows that the amount of Δ9-THC and CBD can be 7 to 12 times above the legal limit of 10 μg/g in hemp seeds	[17]

Note: Supercritical CO_2_ extraction—SC-CO_2_; steam distillation—SD; hydrodistillation—HD; supercritical fluid extraction—SFE; tetrahydrocannabinol—Δ9-THC; Δ9-tetrahydrocannabinolic acid—Δ9-THCA; cannabidiol—CBD; cannabidiolic acid—CBDA; linoleic acid (LA) ω-6; α-linolenic (ALA) ω-3; ethanol—EtOH; methanol—MeOH; C*—the concentration of the saturated solution in contact with the seed particles (g/mL) temperature—T; pressure—P; yield—η; (-) not used.

**Table 2:** Bioactivity of phenolic compounds identified in hemp seeds (*Cannabis sativa* L.).

Bioactive Compounds	Chemical Formula	Uses in the Food Industry	Health Benefits	Reference
Caffeic acid	C_9_H_8_O_4_	Antioxidants	Antitumor, antidiabetic, antiatherosclerotic, anti-Alzheimer’s disease, antibacterial, and antiviral	[23,40,41]
Gallic acid	C_7_H_6_O_5_	Food additive, inhibits oxidation and rancidity of oils and fats	Antioxidant, antimicrobial, anti-inflammatory, anticancer, cardioprotective, gastroprotective, and neuroprotective effects	[23,42,43]
Rosmarinic acid	C_18_H_16_O_8_	Antioxidant; ideal alternative to sulfur dioxide in wine fermentation; could extend the shelf life of pork from 4 to 8 days by inhibiting pH	Anti-inflammatory, antioxidant, antidiabetic, antiviral, antitumor, neuroprotective, hepatoprotective	[23,44]
*p*-OH-benzoic acid	C_7_H_6_O_3_	Preservative	Antimicrobial, antialgic, antimutagenic, antiestrogenic, hypoglycemic, anti-inflammatory, antiplatelet, nematicide, antiviral, antioxidant	[23,45]
Ferulic acid	C_10_H_10_O_4_	Food additive, prevents lipid peroxidation	Antitumor, antidiabetic, cardiovascular and neurodegenerative protector	[23,45]
3,4-dihydroxybenzoic acid	C_7_H_6_O_4_	Antioxidants	Antitumor (prostate, colon)	[23,46]
*p*-coumaric acid	C_9_H_8_O_3_	Natural additive, decreases the peroxidation of low-density lipoproteins (LDL)	Antioxidant, anticancer (lung, colon), antimicrobial, antiviral, anti-inflammatory, antiplatelet, anxiolytic, antipyretic, analgesic and antiarthritic, antidiabetic, antiobesity, antihyperlipidemic, and antigout	[23,47,48]
Syringic acid	C_9_H_10_O_5_	Antioxidants	Antioxidant, antimicrobial, anti-inflammatory and antiendotoxic, neuro- and hepatoprotective, antitumor	[23,49]
Quercetin	C_15_H_10_O_7_	Natural coloring and preservatives	Cardiovascular and neurological protector	[23,50]
Luteolin	C_15_H_10_O_6_	Preservative	Antitumor, helps regulate skin aging and inflammation, reduces fibrosis and inflammation in the liver, antioxidant, antihypertensive, antidiabetic, antiasthmatic, and antiviral	[23,51]
Catechins	C_15_H_14_O_6_	Increases absorption of healthy functional foods	Antimicrobial, antiviral, anti-inflammatory, anti-allergenic, and anti-carcinogenic	[23,52]
Naringenin	C_15_H_12_O_5_	Nutraceutical values	Antioxidant, antitumor, antiviral, antibacterial, anti-inflammatory, anti-adipogenic, hepatoprotective, and cardioprotective	[23,53,54]
Isorhamnetin	C_16_H_12_O_7_	Potential bioactive compound	Antioxidant, anti-inflammatory, and antimicrobial	[23,55]
Resveratrol	C_14_H_12_O_3_	Antioxidants	Anti-inflammatory, anti-carcinogenic, cardioprotective, vasorelaxant, phytoestrogenic, and neuroprotective	[23,56]
Apigenin	C_15_H_10_O_5_	Natural pigment	Antioxidant, anti-inflammatory, anticarcinogenic, antigenotoxic, antiallergic, neuroprotective, cardioprotective, and antimicrobial	[23,57]

**Table 3:** Bioactivity of terpenes identified in hemp seeds (*Cannabis sativa* L.).

Active Compounds	Chemical Formula	Flavor	Uses in the Food Industry	Health Benefits	Reference
Limonene	C_10_H_16_	Citric	Flavoring agent	Antitumor, antiviral, anti-inflammatory and antibacterial properties	[58,59,60]
Linalool	C_10_H_18_O	Floral, with a hint of spice	Food additive, flavoring agent	Antimicrobial and antifungal properties	[60,61]
Caryophyllene	C_15_H_24_	Woody sweetness, clove spice	Food additive, flavoring agent	Anti-inflammatory properties, anticancer, antimicrobial, antioxidant and analgesic activities	[60,62]
α-Humulene	C_15_H_24_	Distinct from hops	Food additive, flavoring agent	Anti-inflammatory properties	[63]
β-Mircene	C_10_H_16_	Slightly sweet, spicy, earthy and musky aromatic notes	Food additive, flavoring agent	Anxiolytic, antioxidant, anti-aging, anti-inflammatory, and analgesic properties	[20,64,65]
Hexanal	C_6_H_12_O	Green peas, grass	Natural antioxidant in various types of meat products	Antimicrobial activity	[66,67]
Octane	C_8_H_16_O	Green, citrus	Production of flavors for the food industry	Antifungal activity, has action in controlling essential tremors (ET) and other types of involuntary neurological tremors	[68,69]
Benzaldehyde	C_7_H_6_O	Almond, sweet, woody	Flavoring agent, food additive	Antimicrobial properties against M. tuberculosis	[70,71,72,73]
Decanal	C_10_H_20_O	Sweet, citrus, green	Flavoring agent, food additive	Antifungal and antimicrobial properties, inhibits UVB-mediated photoaging	[60,74]

**Table 4:** Bioactivity of phytocannabinoids identified in hemp seeds (*Cannabis sativa* L.).

Bioactive Compounds	Chemical Formula	Food Application	Health Benefits	Effect	Reference
Δ9-THC	C_21_H_30_O_2_	-	Properties in the treatment of neuropathic pain, spasticity, overactive bladder	Psychoactive	[78,81]
CBD	C_21_H_30_O_2_	CBD infused foods	Properties in the treatment of anxiety, movement disorders, and pain, anti-inflammatory	In general, it is safe	[80,82]

## Data Availability

MDPI Research Data Policies at https://www.mdpi.com/ethics.
